# Association between CA-125 and post-extubation respiratory failure: a cohort study

**DOI:** 10.1186/s13054-024-04806-5

**Published:** 2024-01-23

**Authors:** Fataki Lombuli, Tiago Hermes Maeso Montes, Márcio Manozzo Boniatti

**Affiliations:** 1 Postgraduate Program in Cardiology, School of Medicine, Universidade Federal do Rio Grande do Sul, Porto Alegre, RS Brazil; 2 Department of Critical Care, Hospital Nossa Senhora da Conceição, Porto Alegre, Brazil; 3 Universidade La Salle, Canoas, Brazil

To the Editor,

Liberating patients from mechanical ventilation (MV) is a crucial step in the care of critically ill patients. Delayed or unsuccessful weaning from MV is associated with unfavorable clinical outcomes [1]. Identifying patients at a higher risk of weaning failure can facilitate optimal timing for extubation and guide appropriate interventions. There are multiple mechanisms contributing to extubation failure, and inadequate cardiovascular response is one of them [2]. Detecting patients at risk of cardiovascular dysfunction during MV weaning using a cost-effective, widely available, operator-independent, and non-invasive method is desirable. In recent years, CA-125 has emerged as a biomarker for congestion in patients with heart failure. Notably, CA-125 levels appear to remain relatively consistent across a range of left ventricular ejection fractions [3], and they are not significantly influenced by factors like renal function and age. This makes CA-125 a promising alternative to BNP in certain clinical scenarios. The aim of this study was to investigate the potential association between plasma CA-125 levels and the occurrence of post-extubation respiratory failure.

We conducted a prospective cohort study in the intensive care unit (ICU) of a tertiary hospital in Porto Alegre, Brazil, between July 2022 and July 2023. We prospectively enrolled patients aged 18 years or older who had received MV for at least 48 h and were ready for a spontaneous breathing trial (SBT). Patients with malignancy, tracheostomies, or do-not-reintubate orders were excluded. Prior to the SBT, lung ultrasound (LUS) images were acquired by one of the investigators (THMM). A point scoring system was applied to each region and ultrasound pattern: A-lines equated to 0 points, separated B-lines to 1 point, coalescent B-lines to 2 points, and lung consolidation to 3 points [4]. Serum CA-125 detection method: Venous blood was collected before the SBT, placed in a vacuum tube, and sent to the laboratory. The SBT was conducted using a T-tube connected to an oxygen source or with low-pressure support (8 cmH_2_O) and PEEP ≤ 5 cmH_2_O with the same FiO_2_ (≤ 40%). In cases of SBT failure, patients were reconnected to the ventilator and excluded from the study. Patients who successfully completed the SBT were extubated. The primary outcome was respiratory failure within 72 h of extubation, defined as the presence of at least two of the following criteria: respiratory acidosis, oxygen saturation < 90% with FiO_2_ ≥ 50%, respiratory rate higher than 25/min for two consecutive hours, or clinical signs of respiratory fatigue.

During the study period, we enrolled a total of 103 patients who underwent SBT, and their CA-125 levels were collected immediately before initiating the SBT. Among these patients, SBT failed in 17 cases, leading to their return to MV. The final analysis included 86 extubated patients, whose clinical characteristics are detailed in Fig. 1A. Forty-four patients (51.2%) experienced post-extubation respiratory failure. Among the respiratory failure cases, 23 patients (52.3%) received NIV, while 16 patients (36.4%) required reintubation within 72 h. In the univariate analysis, patients in the respiratory failure group exhibited significantly higher median CA-125 values (53.7 U/mL [26.9–92.0]) compared to those in the non-respiratory failure group (29.2 U/mL [18.3–60.1], *p *= 0.009), as illustrated in Fig. 1B. The area under the ROC curve for CA-125 in predicting respiratory failure occurrence was 0.663 (95% CI 0.546–0.779). The optimal cutoff point, determined using the Youden index, was identified as 35.0 U/mL. This cutoff exhibited a sensitivity of 70.5%, specificity of 59.5%, positive predictive value of 64.6%, and negative predictive value of 65.8% for predicting respiratory failure. In a logistic regression model adjusted for CHF, SAPS 3, duration of MV, and LUS score, CA-125 levels exceeding 35.0 U/mL remained significantly associated with the occurrence of respiratory failure (OR 3.474, 95% CI 1.375–8.780; *p *= 0.008). In a post-hoc exploratory analysis that involved a combined evaluation of CA-125 and LUS scores, we observed an association with the incidence of the primary outcome (Fig. 1C). Specifically, patients exhibiting CA-125 > 35.0 U/mL alongside LUS exceeding 6.5 points demonstrated an 81.0% incidence of respiratory failure. In contrast, patients with normal CA-125 and LUS scores showed a markedly lower incidence of 32.0% for respiratory failure.

**Fig. 1:**
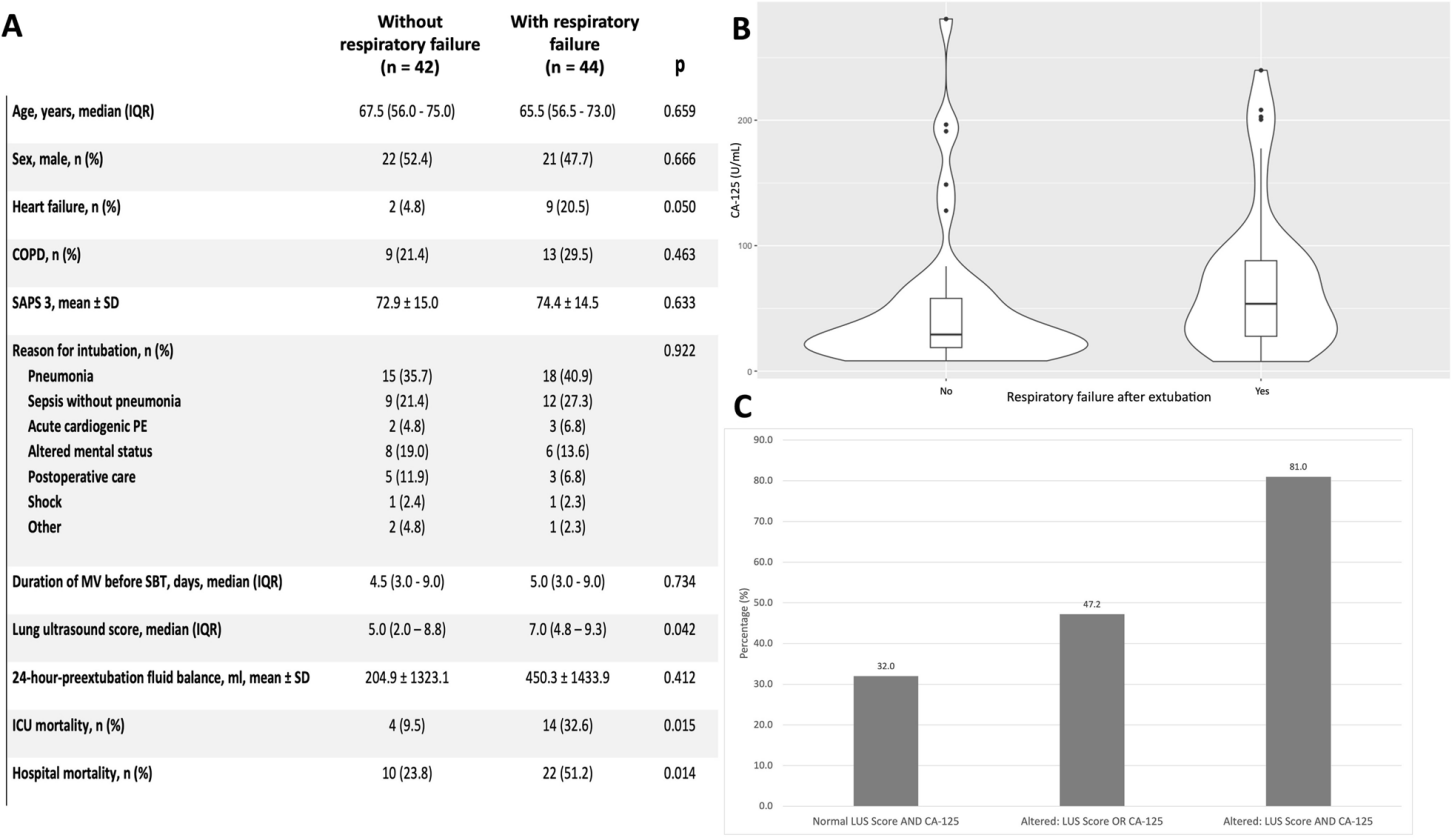
Comparative analysis of clinical characteristics, CA-125, and LUS score in patients with and without post-extubation respiratory failure. **A** Table depicting patients’ main characteristics. **B** CA-125 values in relation to post-extubation respiratory failure. **C** Incidence of post-extubation respiratory failure with combined assessment of LUS score and CA-125

We observed an association between increased CA-125 levels and a higher incidence of respiratory failure among critically ill patients who were extubated following successful SBT. Importantly, these associations remained consistent regardless of the presence of heart failure or the LUS score. While a single CA-125 measurement may provide only moderate accuracy in identifying patients at risk of PERF, our results suggest the potential utility of incorporating CA-125 into routine congestion assessments. This study represents the first exploration of CA-125's application in this specific clinical context. Moreover, our exploratory analysis revealed a potential role for combining CA-125 with the LUS score to evaluate a patient's risk of developing post-extubation respiratory failure.

## Data Availability

The datasets used and/or analyzed during the current study are available from the corresponding author on reasonable request.
